# Changes in the Clinical Characteristics of 62 Patients Who Died from Coronavirus Disease 2019

**DOI:** 10.1155/2020/3280908

**Published:** 2020-08-12

**Authors:** Jingli Chen, Jishi Ye, Hui Li, Zhongyuan Xia, Hong Yan

**Affiliations:** ^1^Department of Anesthesiology, The Central Hospital of Wuhan, Tongji Medical College, Huazhong University of Science and Technology, Wuhan, China; ^2^Department of Anesthesiology, Renmin Hospital of Wuhan University, Wuhan, 430060 Hubei, China; ^3^Department of Anesthesiology, Xiaogan First People's Hospital, Xiaogan, Hubei, China

## Abstract

**Background:**

Since the first reports of severe acute respiratory syndrome coronavirus 2 (SARS-CoV-2) infections in December 2019 in Wuhan, China, the virus has spread to other parts of China and across the world. Although a few studies have assessed the clinical course of coronavirus disease 2019 (COVID-19), the changes in clinical characteristics during disease progression remain unclear.

**Methods:**

We retrospectively analyzed the clinical characteristics of 62 patients who died from COVID-19 at the Central Hospital of Wuhan between January 26 and February 17, 2020. We compared the clinical features on admission and at the last follow-up before death.

**Results:**

Of the 62 patients with COVID-19, 41 (66%) patients were male, and 21 (34%) were female. The median age was 72 years (interquartile range (IQR), 54-88), and 45 (72.5%) patients had preexisting conditions. The median time from symptom onset to the first visit at the clinic was three days, while the median time from symptom onset to death was 18.5 days. During disease progression, the amounts of arterial gases worsened, and liver, renal, and heart dysfunction was observed. Due to the cytokine storm, infection-related biomarkers, including lactic acid, C-reactive protein, and interleukine-6, gradually worsened during hospitalization.

**Conclusion:**

Our findings suggest that during hospitalization, many COVID-19 patients experienced multiple organ dysfunction and cytokine storm. The time from symptom onset to death was only 18.5 days, highlighting the disease's rapid progression. The better understanding of the clinical changes during disease progression might provide further insight into the COVID-19 pathophysiology.

## 1. Introduction

Since December 2019, when the first cases of coronavirus disease 2019 (COVID-19) were reported in Wuhan, Hubei, China, severe acute respiratory syndrome coronavirus 2 (SARS-CoV-2) has rapidly spread to other parts of China and across the world [1, 2]. Subsequently, the National Health Commission of the People's Republic of China has classified pneumonia caused by SARS-CoV-2 as a class “B” infectious disease, while taking preventive and control measures according to class “A” infectious disease guidelines. As of June 24, 2020, 85,098 COVID-19 cases have been confirmed in China, with 4,647 deaths. The number of confirmed cases has reached 9,247,596 worldwide, with nearly 478,000 deaths. Given that the virus has spread to most countries and territories across the globe, the World Health Organization (WHO) has declared COVID-19 as a pandemic [3].

Although the outbreak in China has been largely controlled through prompt and strict measures, the number of SARS-CoV-2 infections has increased dramatically in other countries, posing serious public health threats globally. Epidemiological and clinical data from COVID-19 patients in China provided a valuable reference for the management of domestic cases in other countries [4]. Despite extensive studies on the new disease, many cases are still hospitalized, and their clinical course and outcomes remain unknown. Although a few studies have addressed the clinical course of COVID-19 cases that died of the disease, most studies focused on clinical data on admission or immediately before death. The changes in clinical characteristics during hospitalization could unveil previously unknown aspects of the disease.

Biochemical indicators, including routine blood markers, renal and liver function markers, myocardial enzymes, interleukin-6 (IL-6), procalcitonin, and blood gas indicators, are widely used to monitor changes in the physiological indexes of patients and guide clinical decision making. In this study, we retrospectively reviewed the clinical characteristics of 62 patients who died from COVID-19 between January 26, 2020, and February 17, 2020, at the Central Hospital of Wuhan, Hubei, China.

## 2. Methods

### 2.1. Study Design and Patients

In this retrospective, single-center study, we analyzed the clinical features of 62 patients who died from COVID-19 pneumonia from January 26 to February 17, 2020, at the Central Hospital of Wuhan, Hubei, China. All patients were diagnosed with COVID-19 pneumonia and were tested positive for SARS-CoV-2 with quantitative real-time PCR (qRT-PCR) on respiratory tract samples. The study was reviewed and approved by the Ethics Committee of the Central Hospital of Wuhan (No. 2020-54). The requirement for informed consent was waived by the Ethics Commission, as described previously [5].

### 2.2. Data Collection

Clinical, epidemiological, and laboratory data from the electronic medical records were independently collected and reviewed by CJL and YJS using a customized data collection form to ensure the accuracy of the data.

Using the Chinese Center for Disease Control and Prevention (CDC) recommended Kit (Innovita, Beijing, China), swab specimens from the upper respiratory tract were collected and tested for SARS-CoV-2 nucleic acid. qRT-PCR analyses were performed at the Department of Laboratory Medicine of the Central Hospital of Wuhan following a previously described protocol.

Besides SARS-CoV-2 detection, patients underwent routine blood examination, as well as renal and liver function, myocardial enzyme, C-reactive protein (CRP), D-dimer, lactic acid, IL-6, procalcitonin (PCT), and blood gas tests. These analyses were also conducted at the Department of Laboratory Medicine of the Central Hospital of Wuhan. In this study, we analyzed and compared clinical data on admission and the last follow-up examination before death.

### 2.3. Statistical Analysis

Continuous variables were expressed as means ± standard deviation (SD) or median and interquartile range (IQR), whereas categorical variables were presented as numbers and percentages (%). We used Student's *t*-test to compare the differences between groups. *P* values < 0.05 were considered statistically significant. All statistical analyses were performed using SPSS, version 19.0.

### 2.4. Role of the Funding Source

The funder of the study had no role in the study design, data collection, data analysis, result interpretation, or manuscript writing. The corresponding authors (XZY and YH) had full access to all the data and had final responsibility for the decision to submit for publication.

## 3. Results

In this study, we analyzed the clinical data of 62 patients who died from COVID-19 at the Central Hospital of Wuhan from January 26 to February 17, 2020. Forty-one (66%) patients were male, and 21 (34%) were female. The median age of the patients was 72 years (IQR, 54-88; range, 35-99; Table 1). A total of 45 patients (72.5%) had underlying diseases, including cardiovascular and cerebrovascular diseases (*n* = 33; 53%), respiratory diseases (*n* = 5; 8%), endocrine system diseases (*n* = 13; 21%), cancer (*n* = 2; 3%), and digestive diseases (*n* = 5; 8%). Additionally, two patients developed fever and cough after surgery for rectal cancer and lung cancer, respectively. Twenty-one patients (34%) had a history of exposure to the Huanan Seafood market. The most common symptoms on admission were fever (84%) and cough (66%), followed by polypnea (45%) and sputum production (40%). Less common symptoms on admission included myalgia, decreased appetite, fatigue, diarrhea, headache, pharyngalgia, chest pain, nausea, vomiting, and dizziness. Of the 62 patients, 42 patients (68%) were classified as severe cases on admission, and the remaining 20 patients (32%) were classified as critical cases. The median time from symptom onset to the first visit at the clinic was three days (IQR, 0-6). The median time from symptom onset to death was 18.5 days (IQR, 12-23).

All confirmed COVID-19 patients were treated in isolation. As shown in Table 2, all patients received oxygen therapy during hospitalization. Fifty-six patients (90%) were administered antibiotics (including moxifloxacin, meropenem, imipenem, and cilastatin sodium), and 39 (63%) received antiviral treatment (including oseltamivir, interferon-*α*, ganciclovir, and lopinavir). Only six (10%) patients were treated with corticosteroids (including methylprednisolone sodium succinate, methylprednisolone, and dexamethasone), while 13 patients (21%) were treated with intravenous infusion of immunoglobulin. Due to disease progression, 13 (21%) patients received invasive mechanical ventilation, and six (10%) underwent continuous renal replacement therapy (CRRT). Due to a shortage of medical equipment, only three patients (5%) were treated with extracorporeal membrane oxygenation (ECMO).

A comparison of laboratory findings on admission and at the last follow-up before death is shown in Table 3. In contrast to erythrocyte and platelet counts, the numbers of lymphocytes and neutrophils decreased significantly during disease progression (*P* = 0.023 and *P* = 0.015, respectively). Similarly, the amounts of arterial gases worsen during disease progression. Lactic acid, PaO_2_, and pH values at the last follow-up were significantly worse than those on admission (*P* = 0.022, *P* = 0.013, and *P* = 0.042, respectively). Moreover, the levels of the coagulation marker D-dimer profoundly increased during hospitalization (*P* = 0.001). However, we found no significant changes in the activated partial thromboplastin time and prothrombin time (*P* = 0.065 and *P* = 0.054, respectively). Moreover, markers of liver, renal, and heart function worsen between admission and last follow-up. Notably, alanine transaminase, creatinine, blood urea nitrogen, and cardiac troponin I levels increased significantly during disease progression, whereas albumin levels decreased (*P* = 0.001, 0.001, 0.026, 0.001, and 0.034, respectively). The levels of the infection-related biomarkers CRP and IL-6 were significantly increased during hospitalization (*P* = 0.034 and *P* < 0.001, respectively). However, procalcitonin levels did not change significantly (*P* = 0.125).

## 4. Discussion

In this retrospective study, we investigated the changes in the clinical features of 62 COVID-19 patients hospitalized at the Central Hospital of Wuhan before death. The Central Hospital of Wuhan is the epicenter of this epidemic in China and the site of the worst losses, and elucidating the changes on the clinical characteristics of our patients could provide further insight into the pathophysiology of COVID-19.

SARS-CoV-2, the causative agent of COVID-19, is a highly infectious virus [6]. The viral gene encoding the spike (S) protein can be used for virus typing, while the nucleocapsid (N) protein encapsidates the viral genome and can be used as a diagnostic antigen [3]. Through a comparative analysis of the viral genome, it was speculated that the natural hosts of SARS-CoV-2 might be pangolins and other wild animals [7]. It is also likely that there are unknown intermediate hosts for bat-to-human transmission. The number of confirmed COVID-19 cases and COVID-19-related death is increasing rapidly worldwide. According to the real-time outbreak data released by Johns Hopkins University in the United States, the cumulative number of confirmed cases outside China has exceeded 200,000 as of March 24. At data cutoff for this study, the global mortality rate of COVID-19 was 4.4%. Although the mortality rate of COVID-19 is not as high as that of SARS or MERS, the number of people infected with SARS-CoV-2 is far higher than the number of people infected with SARS-CoV or MERS-CoV. Therefore, the better understanding of the clinical features of COVID-19 is of high clinical importance.

In this study, our cohort consisted of more men than women, which, according to the findings by Mo et al. [8], showing that female COVID-19 exhibit different clinical characteristics from male patients, might have influenced our results. Notably, women reportedly have relatively milder symptoms and a longer incubation period than men, suggesting a stronger antiviral immunity. The median age of patients was 72 years, and 72.5% of patients had underlying diseases. These data indicate that both age and comorbidities are critical factors associated with death. All patients were classified as severe or critically ill at admission, suggesting that early intervention is crucial for the disease outcome.

Considering the shortage of medical resources in Wuhan early during the epidemic, we collected data regarding the time from symptom onset to the first visit at the clinic. Although most patients visited the hospital three days after the onset of symptoms, some patients had to wait for 10-15 days to see a doctor. The time between symptom onset and death was only 18.5 days, reflecting the severity of the disease and corroborating the findings of Wang et al. [9] showing that the time between illness onset and disease progression was 7-13 days. Given the limited data availability, we did not explore risk factors associated with in-hospital death. Given that viral pneumonia was often accompanied by bacterial infections, most of the patients received antibiotics. Nevertheless, many patients died of sepsis due to poor health and the development of drug resistance. Previous studies have reported that boosting the patients' immune system might improve the outcomes of COVID-19 patients [10]. However, due to the high cost and limited availability, only 21% of these patients received immunoglobulin. We believe that early immunotherapy intervention might have improved the survival rate of these patients. Currently, COVID-19 patients are treated with antiviral agents (chloroquine and hydroxychloroquine), corticosteroids, antibodies, and convalescent plasma transfusion [11]. However, there are no specific SARS-CoV-2-targeting agents thus far. Considering the time required for the preclinical and clinical development of effective SARS-CoV-2-targeting agents, the interim research data might guide the current urgent demands for therapy.

We found that in most COVID-19 patients, lymphocyte and neutrophil counts decreased during hospitalization. It is widely believed that SARS-CoV-2 spreads through respiratory particles, causing cytokine storms and decreasing the number of neutrophils and lymphocytes. Given that COVID-19 is a new disease and is not fully understood, it was impossible to test novel biomarkers associated with disease progression. Additionally, it would be impractical to test for other chemokines, such as IL-1 and IL-10, considering that medical resources are scarce. CRP, IL-6, and PCT are commonly used for the diagnosis of various infectious diseases. The combined detection of IL-6, PCT, and CRP can provide a differential diagnosis of early bacterial and viral infections. Although PCT levels did not change during disease progression, CRP and IL-6 levels increased, reflecting increased inflammation.

Likely due to the cytokine storm, several infection-related biomarkers, including lactic acid, CRP, and IL-6, gradually worsened during hospitalization. These biomarkers can reflect not only the degree of inflammation but also the prognosis of the patients. Meanwhile, some patients exhibited signs of organ dysfunction, such as decreased albumin and increased alanine transaminase, creatinine, blood urea nitrogen, and cardiac troponin I. Therefore, early intervention to prevent organ dysfunction and reduce cytokine storm in severe and critical cases are of great importance. Importantly, Luo et al. [12] found that tocilizumab (TCZ), a monoclonal antibody against IL-6, benefited COVID-19 patients by alleviated inflammation. For patients with septic shock and do not benefit from vasopressor therapy, short-term treatment with low doses of glucocorticoids is recommended [13]. However, given the complexity of COVID-19 and the dysfunction in the host immune system, it is crucial to balance the potential risks and benefits of anti-inflammation therapies.

Our study had some limitations. First, it was a descriptive, single-center study based on a small cohort, and potential biases cannot be excluded. Secondly, we did not assess for risk factors associated with COVID-19 mortality. Third, the clinical data for many patients were incomplete, limiting the power of our analyses.

In conclusion, we analyzed the clinical data of patients who died from COVID-19 and found that they were more likely to be older men with underlying diseases. During hospitalization, many patients experienced multiple organ dysfunction and cytokine storm. In this cohort, the time from symptom onset to death was only 18.5 days, highlighting the rapid disease progression. The elucidation of the changes in the clinical characteristics during disease progression may improve the understanding of the clinical manifestations of COVID-19.

## Figures and Tables

**Table 1:** Patient characteristics.

	Patients (*n* = 62)
Age (years)	72 (IQR: 54-88)
Sex	
Male	41 (66%)
Female	21 (34%)
Underlying diseases	45 (72.5%)
Cardiovascular and cerebrovascular diseases	33 (53%)
Respiratory disease	5 (8%)
Endocrine system diseases	13 (21%)
Digestive disease	5 (8%)
Malignant tumor	2 (3%)
Exposure history	21 (34%)
Fever	52 (84%)
Cough	41 (66%)
Polypnea	28 (45%)
Sputum	25 (40%)
Myalgia	23 (37%)
Decreased appetite	23 (37%)
Fatigue	15 (24%)
Diarrhea	12 (19%)
Headache	11 (18%)
Pharyngalgia	9 (15%)
Chest pain	9 (15%)
Nausea or vomiting	8 (13%)
Dizziness	7 (11%)
Time from symptom onset to the first visit at the clinic (days)	3 (IQR: 0-6).
Time from symptom onset to death at the hospital (days)	18.5 (IQR: 12-23)
Disease severity	
Mild	0 (0%)
Normal	0 (0%)
Severe	42 (68%)
Critical	20 (32%)

Note: According to the COVID-19 diagnosis and treatment protocol (trial version 7) published by the National Health Commission of China, the clinical types of COVID-19 are mild, normal, severe, and critical.

**Table 2:** Patients' therapy.

Treatment	Patients (*n* = 62)
Oxygen therapy	62 (100%)
Antibiotics	56 (90%)
Antiviral treatment	39 (63%)
Noninvasive mechanical ventilation	49 (79%)
Invasive mechanical ventilation	13 (21%)
CRRT	6 (10%)
ECMO	3 (5%)
Corticosteroids	6 (10%)
Intravenous immunoglobulin	13 (21%)

**Table 3:** Laboratory tests.

Routine blood test	On admission (*n* = 62)	Last follow-up (*n* = 62)	*P* value
Erythrocytes (×10^9^; normal range: 3.5-5.0)	4.22 (2.35-5.34)	3.97 (2.23-5.4)	0.325
Lymphocytes (×10^9^; normal range: 0.8-4.0)	0.77 (0.13-4.22)	0.58 (0.16-3.38)	0.023
Neutrophils (×10^9^; normal range: 2.0-7.5)	4.25 (3.11-7.86)	3.56 (2.59-6.68)	0.015
Platelets (×10^9^; normal range: 100-300)	203.5 (109.2-286.3)	209.3 (110.9-265.3)	0.214
Blood gas analysis	On admission (*n* = 62)	Last follow-up (*n* = 62)	
PH value (normal range: 7.35-7.45)	7.37 (6.45-7.59)	7.12 (6.23-7.47)	0.042
PaCO_2_ (mmHg, normal range: 35-45)	42.0 (29.0-56.0)	48 (27.0-68.0)	0.084
PaO_2_ (mmHg, normal range: 80-100)	82.0 (55.0-95.0)	65.0 (38.0-78.0)	0.022
Lactic acid (mmol/L, normal range: 0.5-2.2)	2.1 (0.8-10)	3.5 (0.8-14.2)	0.013
Coagulation function	On admission (*n* = 62)	Last time (*n* = 43)	
Activated partial thromboplastin time (s; normal range:25-37)	30.4 (22.3-45.5)	36.7 (24.6-55.3)	0.065
Prothrombin time (s; normal range: 11-14)	11.6 (9.9-23.6)	13.2 (11.2-43.9)	0.054
D-dimer (mg/L; normal range: <0.2)	1.58 (0.1-102.7)	6.48 (0.35-463.2)	0.001
Biochemical indicators			
Albumin (g/L; normal range: 38-48)	32.6 (24.1-45.1), *n* = 62	27.9 (22.7-56.4), *n* = 49	0.034
Alanine transaminase (U/L; normal range: 0-40)	17.5 (4.4-1524), *n* = 62	225.6 (5.9-645.9), *n* = 49	0.001
Creatinine (*μ*mol/L; normal range: 50-120)	66.4 (36.7-1422.5), *n* = 62	36.5 (35.2-1303), *n* = 54	0.001
Blood urea nitrogen (mmol/L normal range: 3.1-9.5)	5.37 (2.44-48.21), *n* = 62	8.26 (4.08-72.42), *n* = 54	0.026
Cardiac troponin I (ng/mL; normal range: <0.1)	0.025 (0.005-1.38), *n* = 62	0.04 (0.004-4.75), *n* = 40	0.001
Infection-related biomarkers			
Procalcitonin (ng/L; normal range: <0.5)	0.47 (<0.05-7.16), *n* = 62	0.63 (0.06-100), *n* = 34	0.125
C-reactive protein (mg/L; normal range: 0.8-8)	2.55 (0.1->20), *n* = 62	4.68 (0.5->20), *n* = 35	0.034
Interleukin-6 (pg/mL; normal range: 56.37-150.33)	22.6 (2.21-92.9), *n* = 36	290.6 (24.42-684.3), *n* = 17	<0.001

## Data Availability

The datasets analyzed during the current study are available from the corresponding author on reasonable request.
